# Trends in access to anti‐malarial treatment in the formal private sector in Uganda: an assessment of availability and affordability of first‐line anti‐malarials and diagnostics between 2007 and 2018

**DOI:** 10.1186/s12936-021-03680-8

**Published:** 2021-03-10

**Authors:** Denis Kibira, Anthony Ssebagereka, Hendrika A. van den Ham, Jimmy Opigo, Henry Katamba, Morries Seru, Tim Reed, Hubert G. Leufkens, Aukje K. Mantel-Teeuwisse

**Affiliations:** 1 Utrecht Centre for Pharmaceutical Policy and Regulation, Utrecht University, Universiteitsweg 99, 3584 CG Utrecht, The Netherlands; 2 Coalition for Health Promotion and Social Development (HEPS-Uganda), Plot 351A, Balintuma Road, Namirembe Hill, Kampala, Uganda; 3 National Malaria Control Division, Ministry of Health, Uganda, Plot 6 Lourdel Road, Wandegeya, Kampala, Uganda; 4 Health Action International, Overtoom 60, 1054 HK Amsterdam, The Netherlands

**Keywords:** Access, Availability, Affordability, Global Fund, Private sector, Antimalarial medicines, Artemisinin-based combination therapies, Diagnostics

## Abstract

**Background:**

Malaria is the single largest cause of illness in Uganda. Since the year 2008, the Global Fund has rolled out several funding streams for malaria control in Uganda. Among these are mechanisms aimed at increasing the availability and affordability of artemisinin-based combination therapy (ACT). This paper examines the availability and affordability of first-line malaria treatment and diagnostics in the private sector, which is the preferred first point of contact for 61% of households in Uganda between 2007 and 2018.

**Methods:**

Cross-sectional surveys were conducted between 2007 and 2018, based on a standardized World Health Organization/Health Action International (WHO/HAI) methodology adapted to assess availability, patient prices, and affordability of ACT medicines in private retail outlets. A minimum of 30 outlets were surveyed per year as prescribed by the standardized methodology co-developed by the WHO and Health Action International. Availability, patient prices, and affordability of malaria rapid diagnostic tests (RDTs) was also tracked from 2012 following the rollout of the test and treat policy in 2010. The median patient prices for the artemisinin-based combinations and RDTs was calculated in US dollars (USD). Affordability was assessed by computing the number of days’ wages the lowest-paid government worker (LPGW) had to pay to purchase a treatment course for acute malaria.

**Results:**

Availability of artemether/lumefantrine (A/L), the first-line ACT medicine, increased from 85 to100% in the private sector facilities during the study period. However, there was low availability of diagnostic tests in private sector facilities ranging between 13% (2012) and 37% (2018). There was a large reduction in patient prices for an adult treatment course of A/L from USD 8.8 in 2007 to USD 1.1 in 2018, while the price of diagnostics remained mostly stagnant at USD 0.5. The affordability of ACT medicines and RDTs was below one day’s wages for LPGW.

**Conclusions:**

Availability of ACT medicines in the private sector medicines retail outlets increased to 100% while the availability of diagnostics remained low. Although malaria treatment was affordable, the price of diagnostics remained stagnant and increased the cumulative cost of malaria management. Malaria stakeholders should consolidate the gains made and consider the inclusion of diagnostic kits in the subsidy programme.

## Background

In 2017, the World Health Organization (WHO) African Region contributed 92% of the estimated 219 million cases of malaria that occurred worldwide [1]. Uganda has the third-highest global burden of malaria cases and contributes to 18% of the malaria cases in East and Southern Africa [2]. Malaria is highly endemic in 95% of Uganda and is the third leading cause of mortality in the country, after neonatal disorders and HIV/AIDS [3]. Pregnant women and children under 5 years of age are the most vulnerable population to malaria [4].

Early diagnosis and treatment of malaria contributes to a reduction in malaria transmission, disease burden and prevents deaths. Universal access to anti-malarial treatment is one of the key strategies of the WHO’s Global Technical Strategy for Malaria 2016–30 aiming to reduce malaria mortality rates by at least 90% by 2030 [5].

In 2006, the WHO recommended the use of artemisinin-based combination therapy (ACT) as the first-line treatment for uncomplicated malaria in the effort to address resistance of *Plasmodium falciparum* to monotherapies and improve treatment outcomes [6]. By 2009, there were concerns about access to quality-assured ACT medicines in many low-income countries (LMICs) that were grappling with a high burden of malaria, especially among the poorest people [7]. Low-cost medicines, for example, chloroquine and sulfadoxine, were widely available and heavily relied on for treatment of uncomplicated malaria, particularly in the private sector outlets [8, 9]. Unfortunately, however, these medicines had gradually become ineffective against malaria parasites. In addition, non-artemisinin-based therapies, artemisinin monotherapies, and low-quality artemisinin-based combinations were widely available [10].

The private sector plays a key role in the fight against malaria [11–13]. In Uganda, the private sector is the preferred first point of contact for about 61% of households [14]. One of the key barriers of access to quality assured anti-malarial treatment is the high price for ACT medicines and malaria Rapid Diagnostic Tests (RDTs) [15]. Price is a major contributor to limited access to medicines because of its impact on affordability, especially for poor people [16]. By 2009, there were few available brands of ACT medicines in the private sector, most of them unaffordable. For example, it was not uncommon to find a dose of a quality-assured, first-line ACT medicines in private sector outlets retailing at six to 21 times higher than the most commonly sold anti-malarial monotherapy regimens (often non-artemisinin-based in composition) [17].

Furthermore, there were equity concerns; children treated for malaria in the public sector were significantly more likely to receive ACT medicines than those treated in the private sector facilities [7].

As a result, initiatives such as the Global Fund’s (GF) Affordable Medicines Facility-malaria (AMFm) pilot in 2011 and later the private sector co-payment mechanism (CPM) in 2013 were implemented to improve access to ACT in the private sector through subsidies. Subsidies supported by interventions to improve demand are important for improving the availability and affordability of ACT medicines in the private-for-profit sector [18]. The AMFm pilot comprised of three components to promote the appropriate use of ACT in the public, private-for-profit and private-not-for-profit sectors. These components were: price reductions through negotiations with prequalified ACT medicines manufacturers to offer 90% price subsidy to selected importers, support of interventions, such as behaviour change communication and establishment of price monitoring [19]. In December 2013, the AMFm programme was rebranded to the CPM to focus only on the private sector [20] and offered a 70% price subsidy for ACT medicines, but not for RDTs [21]. These efforts were expected to culminate into a trickledown effect of reduced ACT medicines prices in the anti-malarial supply chain to the end user. Details of the CPM are outlined elsewhere [21, 22]. Table 1 lists the interventions conducted.

**Table 1:** List of interventions for increased access to ACTs in Uganda

Years	Intervention
2005	National policy on malaria treatment recommends ACTs as first line treatment for malaria case management following emergence of parasite resistance to the medicines such as chloroquine and sulfadoxine/pyrimethamine [23]
2010	WHO introduces *Test and Treat* policy for malaria. With universal access to parasitological diagnosis of malaria possible using rapid diagnostic tests (RDTs), WHO recommended that all cases of suspected malaria should have a test to confirm diagnosis [24]
2011	GF introduces AMFm programme in Uganda: 80% subsidy for anti-malarials Monitoring of anti-malarial availability and prices
2015	GF introduces CPM programme in Uganda: 70% subsidy for anti-malarials Monitoring of anti-malarial availability and prices Training of health workers Behaviour change communication programme for community

Whereas the report on the effect of the multi-country (including Uganda) AMFm pilot was published in 2012 under the ACTwatch group [18–22, 25, 26], there is a paucity of evidence relating to access to anti-malarials prior to and beyond the end of the AMFm pilot. Between 2008 and 2018, the Global Fund disbursed a cumulative total of USD 414,963,377 for strategies to improve access to anti-malarial commodities in Uganda [27]. Despite these continued efforts to support access to anti-malarial commodities in Uganda, there are limited data to show whether the initially observed positive effects were sustained in later years. This paper examines trends in availability and affordability of first-line malaria treatment and diagnostics in Uganda between 2007 and 2018.

## Methods

A cross-sectional design with quantitative methods was used to assess the availability, price and affordability of ACT medicines and diagnostics using a standardized methodology co-developed by the WHO and Health Action International (HAI), adapted for anti-malarials and diagnostics [28]. This methodology prescribes a minimum of 30 facilities per sector and has been validated and used widely [29–32].

Annual studies were conducted prior to 2016 and in 2018 while quarterly studies were conducted for the period of October 2016 to Dec 2017. However, the 2007–2010, and 2014–2015 surveys did not measure the availability and prices of RDTs.

### Selection of survey areas and medicine outlets

Data was collected from six regions in the country: Central, Eastern, Western, Northern, Southern and West Nile. The regions were selected as a realistic representation of the geographical characteristics of the country. One major town within one day’s car travel from the capital city was selected from each of the regions. The facilities surveyed were randomly sampled.

In each region, the main public hospital was used as an anchor from which private medicine outlets were selected within 3 h travel of the hospital. The final list of outlets surveyed was approved by the National Malaria Control Programme (NMCP). Only registered drug outlets were considered in the survey. The official list of registered pharmacies from the national medicines’ regulatory agency (National Drug Authority) was used to locate, select and verify registration status. Table 2 shows the sample frame for the surveys.

**Table 2:** Sample for retail outlets between 2007 and 2018

Year	2007	2008	2009	2010	2012	2013	2014	2015	2016	2017	2018
Sample size	30	30	30	30	120	120	30	30	450	450*	30
Surveyed	23	35	33	32	185	189	41	37	477	494*	38

* Quarterly surveys were conducted in 2017

### Surveyed ACT medicines and RDTs

A list of ACT medicines and RDTs was developed including the formulation and strength of each medicines. This paper however focuses on artemether/lumefantrine (A/L), adult 24 tablet pack, which is the first line recommended treatment for uncomplicated malaria [1] and also has the largest market share of ACT medicines in Uganda and was the main first line anti-malarial consistently surveyed in all the years [9, 33].

### Data collection

Data collection teams of six persons per region worked in pairs composed of a pharmacist and social scientist. The teams were coordinated by a regional supervisor who was a pharmacist by profession. Prior to data collection, survey personnel participated in the training. One key respondent was targeted for the survey at health facility level; either the in-charge, attendant, owner or any suitable person delegated by the in-charge/owner. The respondents included medical doctors, clinical officers, nurses, nursing assistants, midwives, pharmacists and dispensers.

For each medicine available at the outlet on the day of the visit, data collectors recorded: the brand names of two products; the highest and lowest priced medicine in Uganda Shillings (UGX); the strength and price found. Any discounts or other considerations affecting the price to patients were documented. Originator medicines were included if found available.

Each regional supervisor checked all the filled data collection forms for completeness, legibility and consistency, at the end of each day and validated the data collected in 10% of the sample outlets in the region. In addition, the survey manager checked all the data collection forms for completeness and consistency. Data entry was done into an expanded standardized WHO/HAI International Price workbook with multiple quality assurance processes; for example, double entry of the unit prices and use of the workbook’s inbuilt data checking process.

### Data analysis

Computation of medicine availability, prices and affordability was done according to the WHO/HAI methodology [28]. The availability of A/L was calculated as the percentage of sampled medicine outlets where the medicine was found on the day of the visit. For 2017 where quarterly studies were conducted, the annual average was taken.

The median, minimum and maximum patient prices were estimated for A/L and RDT patient prices. Medicine prices obtained during the survey were expressed as ratios relative to a standard set of international reference prices by dividing the median local unit price by the international reference unit price [34]. Medicine price ratios (MPRs) were calculated only if price data from at least four medicine outlets were available. The medicines prices (in UGX) were converted into US dollars (USD) using the prevailing mid-month exchange rates taken from the Bank Uganda website on the first day of data collection [35]. Affordability was assessed by estimating the number of daily wages required for one course of treatment using the daily salary of the lowest-paid government worker (LPGW) in Uganda, for the respective survey year [36].

## Results

### Availability of A/L and RDTs

**Fig. 1:**
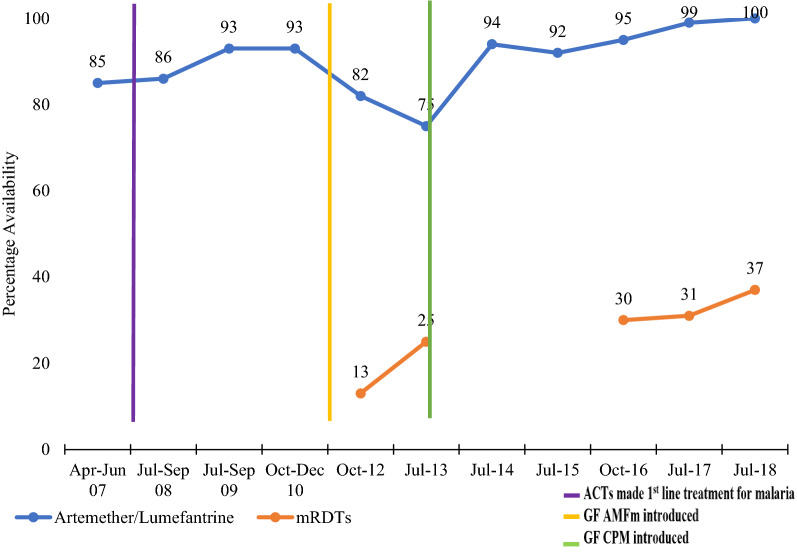
Trends in availability of A/L and mRDTs in the private sector in Uganda

Between 2007 and 2018, the availability of A/L in the private sector facilities gradually increased from 85 to 100%. There was an increase in availability of A/L from 85 to 93% between 2007 and 2009, from which it thereafter dropped from 93 to 75% between 2010 and 2013. However, A/L availability increased from 75 to 94% between 2013 and 2014, then later peaking at 100% in 2017 and 2018. Overall, low availability of RDTs was noted despite the rise from 13% to 2012 to 37% in 2018. Trends in availability of A/L and RDTs are shown in Fig. 1.

### Patient prices and affordability of A/L and RDTs

**Fig. 2:**
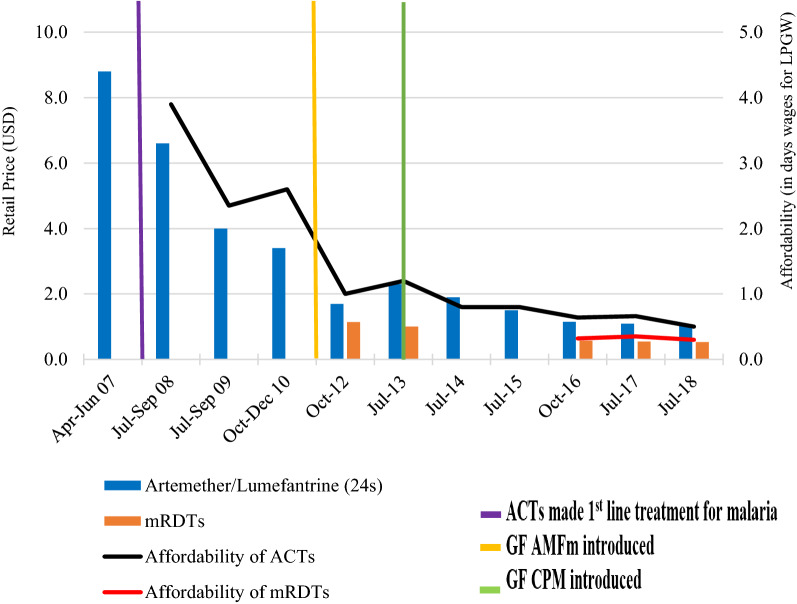
Trends in prices (in USD) and affordability of A/L and mRDTs in private facilities in Uganda

There was a gradual reduction in prices of A/L from a high of USD 8.8 in 2007 to a stable USD 1.1 between 2016 and 2018, while the price of RDTs was mostly consistent at USD 0.5. There was an increment in the prices for A/L from USD 1.7 and USD 2.3 between the end of the AMFm in 2012 and just before the introduction of the CPM in 2013. Affordability of A/L gradually dropped from 4 days’ wages in 2008 to half-a-day’s wages in 2018. Affordability of RDTs improved from about 0.6 in 2012 to about 0.3 days’ wages for LPGW by 2018. Trends in prices and affordability of A/L and RDTs are shown in Fig. 2.

## Discussion

This paper presents trends in the availability, prices and affordability of first line anti-malarial treatment and diagnosis in the private sector in Uganda between 2007 and 2018. There was high availability of first line treatment A/L: at least nine of every ten private medicine outlets surveyed were found with A/L in stock reaching 100% availability in 2018. However, availability of RDTs remained below 40%. There was also a marked reduction in prices of A/L during the period from USD 8.8 to USD 1.1, but price of RDTs remained mostly stagnant at USD 0.5. Affordability of an adult dose of ACT medicine improved from 4 days’ wages to 0.5 days’ wages of the LPGW, and affordability of RDTs remained at about 0.3 day’s wages for LPGW.

Findings from this study are consistent with those reported from a similar programme by ACTwatch that reported an increase in availability of RDTs between 2009 and 2015, but availability remained lower in the private sector compared to the public sector in countries where AMFm programme was administered by the Global Fund [9]. The findings not only focus on RDTs but include ACT medicines, cover a longer period and confirm a sustained effect of the programme in Uganda.

There was a decline in the availability of A/L between 2010 and 2013 dipping below the 80% WHO benchmark in 2013, which may be the result of the high volatile prices of ACT medicines on the market after the introduction of AMFm [21, 37]. After 2013, availability of A/L gradually improved to 100% which could be attributed to consistent supply together with support interventions such as behaviour change communication to improve demand and price monitoring to study changes in the private sector [38]. The changes observed could have been influenced by increased generic competition which led to a reduction in prices of the ACT medicines. It is important to also note that supporting activities to increase awareness about malaria treatment guidelines by health practitioners in the private sector, as well as continuous stakeholder engagements at all levels was helpful [33]. Health system actors particularly in the public sector should therefore learn from and scale up this experience.

Parasitological confirmation particularly with RDTs at community level is important to reduce unnecessary treatment with anti-malarial drugs and to improve the diagnosis of other febrile illnesses. However, availability of RDTs improved slightly over the study period but remained low (below 40%) due to the fact that there were no incentives geared towards making the commodity more available and affordable. Also, contextual aspects such as RDTs being regarded in the private sector as a product sale and not a paid service may have played a role [38]. The high RDT prices push the overall price of malaria diagnosis and treatment to nearly one day’s wages for LPGW. This makes starting treatment almost unaffordable—the WHO benchmark for affordability is 1-day wages [28]—which may lead to suspected malaria patients being treated without confirmatory tests. This is a bottleneck towards successful optimization for the ‘Test, Treat and Track’ Policy for malaria in the private sector [39]. Administering the anti-malarial medicines without a confirmed positive diagnosis may, in the long run, expose the patient to development of resistant strains of the *Plasmodium* as well as the wastage of resources, especially in case the patient does not have malaria [40, 41].

Cumulatively, enormous investments have been made towards malaria control in Uganda, contributing towards improved availability of commodities and the gradual decline of burden of mortality in the country [42–44]. An estimated 90% of the Global Fund grants are annually spent on the procurement of medicines and health commodities [45]. However, disbursement for malaria control interventions was relatively stagnant between 2007 and 2018, with the exception of 2015 when the private sector CPM’s supportive interventions were also implemented to meet the growing demand for the medicines [39, 45]. Consistent and sufficient financing of all recommended strategies is important to ensure elimination of malaria.

The marked reduction in prices of A/L over the review period may warrant further investigation but was largely attributed to global and national efforts aimed at making the products more affordable. In addition, improved access to cheap generics led to increased price competition in the market which improved affordability of treatment of acute malaria. Furthermore, the Ministry of Health of Uganda in 2016 instituted an initiative to regulate the prices of ACT medicines on the private sector market by setting a Recommended Retail Price (RRP) for the ACT medicines (by pack size) which could also have influenced the stability of A/L prices. It should be noted that the prices of ACT medicines remained stable at USD 1.1 between 2016 and 2018, which may further demonstrate that a combination of subsidies, support interventions and price capping policy initiatives could be effective towards ensuring affordability and sustainable access to medicines [46]. However, this RRP was higher than that set earlier on during the AMFm pilot of USD 0.47 for an adult dose of ACT medicine [25].

The anti-malarial market still faces other challenges including uncertainty about the quality of the anti-malarial medicines and RDTs, market speculations at the end of the CPM funding rounds which force hoarding of products by wholesalers and absence of health insurance for majority of the vulnerable populations. In addition, there are concerns about the sustainability of the gains made by the CPM given that the programme is largely donor-funded and there are minimal strategies in place for business continuity in case donor funding ceases, as well as plans to absorb any possible after-shocks [47]. On the other hand, the results observed in this study present opportunities for learning from the private sector that can be utilised for the public sector.

This assessment has been made using a standardized, tested, reliable, and validated WHO/HAI methodology that has been used widely across the world for the measurement of access to medicines [29]. The WHO/HAI methodology uses a cross-sectional design but this assessment provides a longitudinal view of historical trends over a period of 12 years which increases reliability. The assessment is based on several studies conducted annually or quarterly and, therefore, used different samples. This should not be a problem because the standard WHO/HAI methodology recommends thirty outlets in a sector for a survey to achieve enough data points for analysis [29], which was achieved in all years. The findings presented here are not intended to give a full view of the country’s pharmaceutical supply chain but to stimulate policy discussions. The study was conducted in the formal private sector and therefore informal/unlicensed facilities which mainly offer treatment to the majority of the rural population were not included. Also, the study does not explore various supply chain and socio-economic factors which affect availability and affordability.

## Conclusions

Subsidies to the private sector have had a positive impact on the availability and affordability of A/L that should improve malaria management especially among the vulnerable population of pregnant women and children. However, future considerations could be made towards providing subsidies for RDTs in the private sector to improve affordability of the cumulative cost of the test and treatment of malaria.

## Data Availability

The datasets used and/or analysed are available on request from the corresponding author.

## Abbreviations

ACT: Artemisinin-based combination therapy
RDTs: Malaria rapid diagnostic tests
GF: Global Fund
AMFm: Affordable Medicines Facility-malaria
CPM: Private sector co-payment mechanism
A/L: Artemether/lumefantrine
